# Characteristics of patient with borderline personality disorder in a sample of Tunisian out patient

**DOI:** 10.1192/j.eurpsy.2023.2067

**Published:** 2023-07-19

**Authors:** O. Sana, S. Ajmi, B. Olfa, S. Najeh, F. Rim, G. Imen, C. Nada, B. T. Jihen, Z. Lobna, M. Mohamed, M. B. Manel

**Affiliations:** Psychiatry C Department, Hedi chaker University hospital, sfax, Tunisia

## Abstract

**Introduction:**

Borderline personality disorder (BPD) is a real source of suffering for people affected and for those around them.

**Objectives:**

The aim of this study was to describe the socio-demographic and clinical characteristics of out patients with BPD consulting the psychiatry department of Hedi Chaker hospital in sfax tunisia during the period between January 2022 and October 2022.

**Methods:**

It was a descriptive study. The population study consisted of patients diagnosed with BPD (DSM 5) consulting the psychiatry department of the hospital Hédi Chaker of Sfax tunisia during the period from January 2022 to October 2022.

Sociodemographic and clinical data were collected using a predefined form.

All statistical analyses were performed using the SPSS software package v 18.

**Results:**

Among 700 adult patients referred to the psychiatric unit of hedi chaker hospital in sfax from january 2022 to october 2022, 35 patients (5%) were identified as meeting the criteria for BPD.

The average age was about 35 years, 54.3% of the patients were men. They were married in 40% of cases.Only 31.4% of the patients had a regular job.

The presence of a family psychiatric illness was noted in 48.5% of cases and 20% had a childhood psychiatric follow-up .

The rates of sexual abuse, physical abuse, psychological abuse and neglect were 17.1%, 65.7% and 68.6% respectively.

In our sample,48.6% of the patients had attempted suicide and 60% of them had committed self-harm.

The most common means of attempted suicide was phlebotomy.No prior thoughts of suicide were mentioned, all suicide attempts were impulsive. Negative feelings and family conflicts motivated the suicide attempt (37.5% ,56.25%).

Comorbid psychiatric disorder was mentioned in 54.3% and The most frequent comorbid psychiatric disorders was depression (20%).

Pharmacotherapie was used in 88.6% of cases.

**Conclusions:**

The results emphasize on the comorbidities with mainly depressive episodes and a high proportion of suicide attempts and self-harm. Moreover, this study confirms the impact of family conflicts and abuse in the development of this disorder

**Disclosure of Interest:**

None Declared

